# Frailty and COVID 19 Infection in Hospitalized Veterans: A Case Series

**DOI:** 10.1093/geroni/igaa057.3466

**Published:** 2020-12-16

**Authors:** Marlena Fernandez, Valerie Hart, Alma Diaz, Lorena Burton, Victor Cevallos, Jorge Ruiz

**Affiliations:** 1 Miami VA Healthcare System, Miami, Florida, United States; 2 University of Miami / Jackson Health System, Miami, Florida, United States

## Abstract

Frailty, a clinical syndrome characterized by vulnerability to stressors resulting from loss of physiological reserve across multiple systems. In patients with COVID 19 infection, the presence of frailty may place older adults at higher risk for poor clinical outcomes including hospitalizations and mortality. The aim of this case-series study was to describe the characteristics of patients with frailty and COVID-19 who were hospitalized at a VA Medical Center. A VA Frailty Index (VA-FI) was generated at baseline as a proportion of variables from electronic health records. The VA-FI categorized Veterans into non-frail (FI<.21) and frail (FI≥.21). We calculated the VA-FI for Veterans admitted at the time of COVID-19 admission date. We compared the characteristics of frail and non-frail Veterans. A total of 137 veterans were admitted, 96.3% (n=132) were male, mean age 66.81 (SD=13.87) years, and 65.7% (n=90) were frail. When comparing Veterans who were frail versus non-frail, there were no differences in age, race, ethnicity, BMI, rates of cardiopulmonary resuscitation, ICU admissions, use of vasopressors or length of stay. There were significant differences in rates of intubation (frail n=10, vs. non-frail n=0), p = 0.018 and non-invasive respiratory support (frail n=9 vs non-frail n=0), p=.025. There were 13 and 7 readmissions in the frail and non-frail groups respectively. Eleven Veterans died during hospitalization, all of whom were frail. Frailty is associated with poor clinical outcomes in hospitalized Veterans with COVID 19 infection. Recognition of frailty may help to optimize the management of COVID 19 related complications.

